# Professional exposure to basaltic rock dust: assessment by the *Vibrio fischeri* ecotoxicological test

**DOI:** 10.1186/1745-6673-8-23

**Published:** 2013-09-02

**Authors:** Caterina Ledda, Venerando Rapisarda, Massimo Bracci, Lidia Proietti, Matteo Zuccarello, Roberto Fallico, Maria Fiore, Margherita Ferrante

**Affiliations:** 1 Department “G.F. Ingrassia”– Hygiene and Public Health, University of Catania, Catania, Italy; 2 Department of Internal Medicine and Systemic Diseases – Occupational Medicine, University of Catania, Catania, Italy; 3 Occupational Medicine, Department of Molecular Pathology and Innovative Therapies, Polytechnic University of Marche, Ancona, Italy

**Keywords:** *Vibrio fischeri*, Microtox^®^, Basaltic stone, Ecotoxicological test, Ash, Mount Etna

## Abstract

**Background:**

A recent study demonstrates that inhalation of airborne particulate from Mount Etna eruptions may induce fibrotic lung disease. The occupational exposure of construction workers from the Etna area, who excavate building sites and use basalt dust to make mortar, has never been assessed.

**Methods:**

Samples of basalt, volcanic ash, basalt + cement and cement dust were collected on the construction site of a subway tunnel, ground to dust and subjected to the Microtox^®^ solid-phase test to evaluate the toxicity of dust suspensions. Samples were investigated by scanning electron microscopy equipped with energy dispersive X-ray analysis (EDX). Minerals were identified and characterized by their morphology and elemental composition.

**Results:**

The elements found most frequently were C, Na, Mg, Al, Si, K, Ca, Ti, Mn, Fe and O. All four dusts were toxic: basalt and ash were significantly less toxic than basalt + cement and cement, which shared a similar and very high degree of toxicity. Higher Fe, Ca and Mg concentrations were associated with greater toxicity.

**Conclusions:**

The risk related to long-term occupational exposure to various dusts on constructions sites in the Mount Etna area should be further assessed.

## Background

Studies of ash exposure related to volcanic activity among the residents of the Etna area (Sicily, Italy) have shown an increase in the rate of acute respiratory and cardiovascular diseases [1,2] and accumulation of heavy metals in the airways [3]. In a recent study Censi and co-workers [4] demonstrated that inhalation of airborne particulate from Mount Etna eruptions may be responsible for fibrotic lung disease. The possible health effects experienced by construction workers excavating basaltic rock, which forms from volcanic eruptions, have never been investigated.

Considerable effort has been devoted to studying the physical structure [5,6] and chemical composition of volcanic ash [7]. Chemical analysis assists in determining elemental concentration and provides estimates of ash distribution [8,9]. However, chemical data alone do not provide information on all its potential effects on the environment.

Some worker categories, especially those employed in the construction industry, are exposed to basaltic rock dust, volcanic dust and cement. Furthermore cement and basalt dust are often mixed to make mortar.

Cement dust inhalation is associated with an increased prevalence of chronic respiratory symptoms and a reduction of ventilatory capacity [10,11]. In a number of risk assessment studies [12-17] toxicity tests are associated with chemical data in the framework of tiered decision-making, since bioassays provide ecologically relevant information and are rapid and cost-effective screening tools; such tests are however time-consuming and some test organisms require further culture.

The advantages of various test organisms were compared by Hsu et al. [18], who noted that bioassays based on bacteria (such as Microtox^®^) involve a simple procedure, short testing times and are cost-effective. For instance analysis using *Vibrio fischeri*, available in many countries and recommended by international standards (Standard Methods, 1995, ISO/DIS 11348, DIN 38412), is a rapid and economical assay to monitor the toxicity of environmental contaminants, and has been applied to investigate metal plating wastewater [19] and air pollutants [20]. However, limited data are available on the biotoxicity of dust, especially ash and basaltic rocks [21-23].

The aim of this study was to assess the ecotoxicological effects of suspensions of basaltic rock, ash and cement dusts, which are typically found on construction sites in the Etna area, on the natural bioluminescence of the marine bacterium *V. fischeri* (Microtox^®^, AZUR Environmental).

## Methods

### Sampling

Basalt residues (A), volcanic ash (B), mixed basalt and cement (C) and cement (D) were collected on the construction site of a subway tunnel in Catania, Sicily. Basalt is representative of the volcanic and volcaniclastic lithologies of the area; to make the sample more representative the actual dust produced by the excavation work was collected (C and D).

Samples were collected and transported to the laboratory in polyethylene bags. They were dried in an oven at 40°C and stored in polyethylene containers.

### Sample characterization

Dust samples were prepared with gold coating under vacuum, to improve image quality and examined with a scanning electron microscope (SEM) (Cambridge Stereoscan 360) equipped with an energy dispersive X-ray (EDX) system (Oxford Instruments INCA Energy) for semi-quantitative chemical analysis (minimum spot size, 5 μm; working distance, 10 mm; accelerating voltage, 20 kV). Samples were examined at different magnifications for their morphological features and analyzed by EDX to establish their chemical composition. Spectra of 10 points were acquired for each sample.

### *Vibrio fischeri* ecotoxicological test

Two grams of each dust type were extracted with 50 ml Diluent Solution (AZUR Environmental), a specially prepared non-toxic 2% sodium chloride solution. We used a Microtox Model 500 analyzer (SDIx, USA) and carefully performed all the procedures described in the Microtox^®^ standard protocol [24]. Freeze-dried luminescent *V. fischeri* bacteria (NRRL B-11177) were reconstituted and exposed in duplicate to four diluted extracts osmotically adjusted with Microtox osmotic adjusting solution (a specially prepared non-toxic 22% sodium chloride solution). The decrease in bioluminescence induced by exposure to the dusts was measured at 5 and 15 min at a constant temperature of 15°C. Only the 15 min data are reported in this study. All Microtox^®^ data were recorded and analyzed by the online software. Results are expressed as effective concentration 50% (EC_50_) in the 2 g samples. The toxic effect of each sample was assessed as toxicity units (TU), which were calculated as follows:

$$\mathrm{TU}=\left(1/{\mathrm{EC}}_{50}\right)\times 100\%$$

TU represents relative toxicity as described by Kahru *et al.*[25]: <1% = non toxic; 1–40% = toxic; 40–100% = very toxic; >100% = extremely toxic.

To check the reliability of the Microtox^®^ method and reagents, a toxicity test was carried out each day using a phenol aqueous solution (100 mg/L) prior to beginning sample testing. Its results were compared with the Microtox^®^ quality assurance product data. Procedural blanks were also tested to determine whether any toxicity was being contributed by the residual extracts and glassware. No toxicity was detected in the blanks.

### Data analysis

The EDX data were analyzed and standard deviation (SD) was calculated to identify the compounds that were most frequently found in the dust samples.

Student’s *t* test with Bonferroni’s correction was applied with SPSS 20.0 to compare the changes in TU based on the concentrations of the elements found most frequently in the four samples.

## Results and discussions

**Figure 1:**
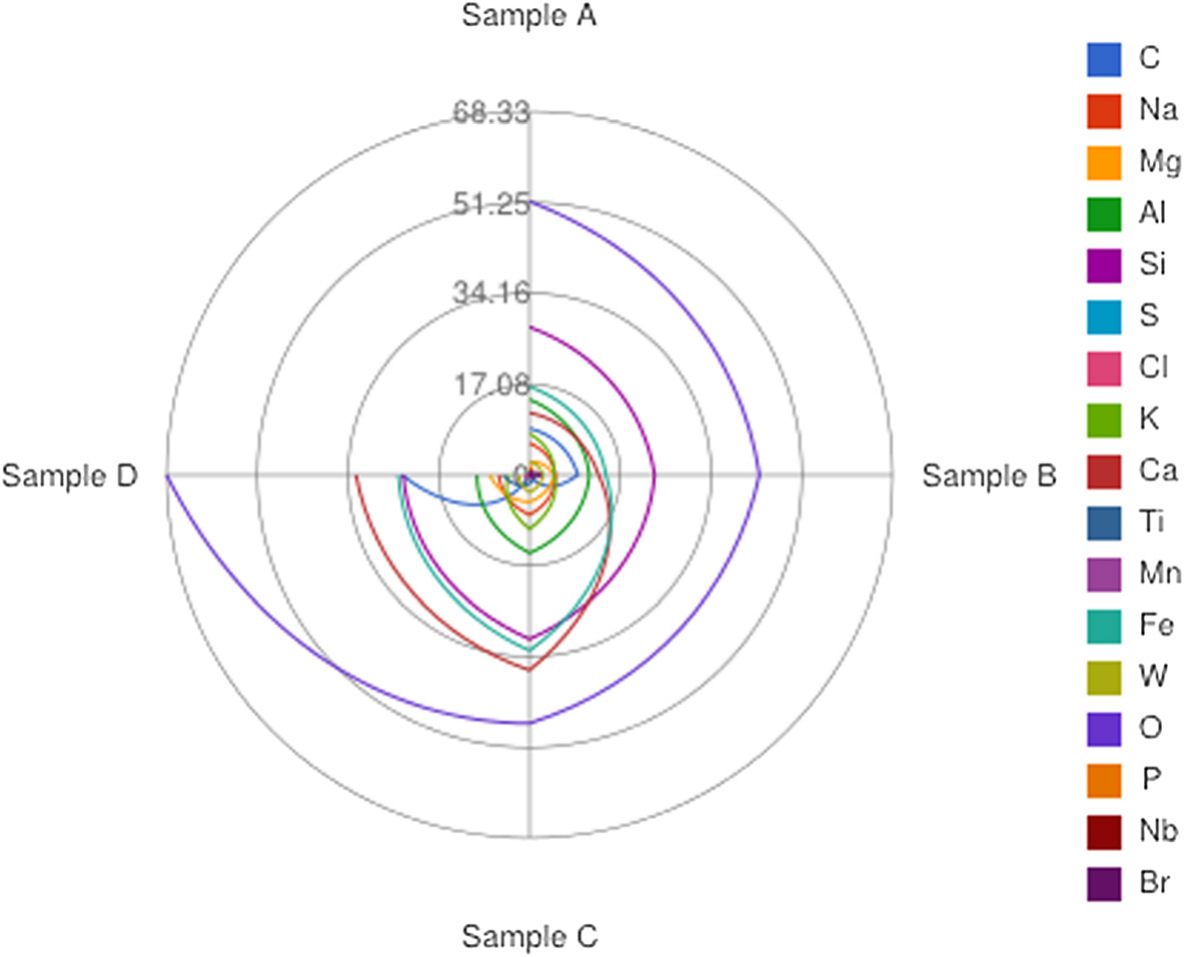
**Concentrations of the elements found more frequently in the four samples (% weight).** Elements are distinguished by color as specified in the figure.

The results of EDX analysis are reported in Table 1 as the mean of 10 spectra per dust type. The per cent weight of the elements detected more frequently in each sample is shown in Figure 1. These elements were: C, Na, Mg, Al, Si, K, Ca, Ti, Mn, Fe and O.

**Table 1:** EDX analysis (% weight)

	***C***	***Na***	***Mg***	***Al***	***Si***	***S***	***Cl***	***K***	***Ca***	***Ti***	***Mn***	***Fe***	***W***	***O***	***P***	***Nb***	***Br***
**Sample A**	8.79	5.98	2.36	14.2	27.79	0.00	0.59	7.80	11.72	0.52	0.60	16.64	2.48	51.42	1.81	0.99	0.00
**Sample B**	9.11	4.86	4.61	11.25	23.54	0.00	0.24	4.58	12.99	0.70	0.00	14.36	2.53	43.26	0.00	0.00	2.11
**Sample C**	0.00	7.51	5.05	14.72	30.82	0.00	0.58	10.20	36.70	1.64	0.00	33.02	3.24	46.71	0.00	0.00	0.00
**Sample D**	24.04	5.78	7.52	9.88	23.80	0.83	0.99	3.79	32.66	5.01	0.61	24.71	1.97	68.33	0.00	0.00	0.00
**P value**	**P < 0.05**	n.s.	n.s.	n.s.	**p < 0.05**	n.s.	n.s.	n.s.	**p < 0.05**	n.s.	n.s.	**p < 0.05**	n.s.	**p < 0.05**	n.s.	n.s.	n.s.

*n.s.* not significant.

The *t* test for paired data with Bonferroni’s correction for multiple comparisons showed significant differences for C, Ca, Fe, O, Si and Mg (p < 0.05) (Table 1).

The Microtox^®^ test results indicated that all dust samples induced acute toxic effects on *V. fischeri* (Table 2). The toxicity measured in the samples of basalt stone dust (A) and volcanic ash (B) was significantly lower than that of the mixed material (C) and of cement (D), which shared a similar high degree of toxicity (close to 100%).

**Table 2:** Microtox^®^ test results: toxicity of the four dust samples

	**TU (%)**
**Sample A**	19.31
**Sample B**	31.22
**Sample C**	**99.05**
**Sample D**	**98.95**

**Figure 2:**
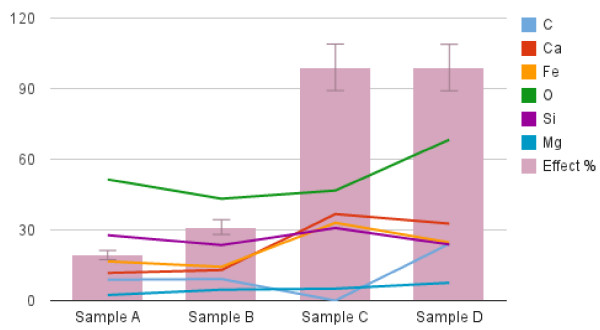
**Profiles of the elements for which student’s *t *test with Bonferroni’s correction provided significant differences and toxic effect (0-100%) of the dust suspensions in relation to their per cent content in the elements found more frequently.** Elements and % effect are distinguished by color as specified in the figure.

Figure 2 demonstrates that rising Ca, Fe and Mg content was associated with increased toxicity, whereas this did not occur with O, C, and Si.

The present findings document the toxic nature of all the dusts studied. Among these, cement appears to be more toxic than basalt dust and volcanic ash. Combining cement with basalt dust does not alter its toxicity.

## Conclusion

The ecotoxicological approach, suggested by Coutand et al. [26], is a new method to assess the occupational risk associated with exposure to dust and ash.

The toxicity of our volcanic ash and basalt dust samples was considerably lower than that of cement and of cement mixed with basalt dust.

Application of the Microtox^®^ ecotoxicological test documented the reactivity of the dusts to which construction workers employed on building sites in the Etna area are consistently exposed. The risk related to their long-term exposure should be further assessed. Our study protocol provides an exhaustive characterization of the potential of the dust to become a respiratory hazard. Health impact studies are essential to make officials and the public aware of the potential health impacts of volcanic emissions and of how public health resources should be allocated.

## Abbreviations

EC50: Effective concentration; EDX: Energy dispersive X-Ray; SEM: Scanning electron microscope; TU: Toxicity unit.

## Competing interests

To the best of our knowledge, no conflict of interest, financial or other, exists.

## Authors’ contributions

All authors read and approved the final manuscript. CL-principal investigator, study design, laboratory support, statistical analysis, paper preparation; VR-study design, sampling, data interpretation, paper preparation; MB-text revision; LP-data interpretation; MZ-laboratory support; RF-text revision; MFi-data collection and interpretation; MFe-group coordination, study design, text revision.

## Authors’ information

CL- Bachelor of Science, Medical laboratory scientist; VR- MD, PhD, Occupational Physician; MB- MD, PhD, Occupational Physician; LP- MD, Assistant Professor of Occupational Medicine; MZ- Chemical technician; RF-MD, Professor of Hygiene; MFi- MD, PhD, Assistant Professor of Hygiene; MFe- MD, Professor of Hygiene.
